# A rat model to investigate quality of recovery after abdominal surgery

**DOI:** 10.1097/PR9.0000000000000943

**Published:** 2021-06-30

**Authors:** Juan P. Cata, Miguel Patiño, Michael J. Lacagnina, Jiahe Li, Aysegul Gorur, Ruben Agudelo-Jimenez, Bo Wei, Carin A. Hagberg, Patrick M. Dougherty, Imad Shureiqi, Peiying Yang, Peter M. Grace

**Affiliations:** a Department of Anesthesiology and Perioperative Medicine, The University of Texas MD Anderson Cancer Center, Houston, TX, USA; ^b^Department of Anesthesiology, Massachusetts General Hospital, Boston, MA, USA; c Laboratories of Neuroimmunology, Department of Symptom Research, The University of Texas MD Anderson Cancer Center, Houston, TX, USA; d Department of Anesthesiology and Perioperative Medicine, University of Washington, Seattle, WA, USA; Departments of e Integrative Medicine Research; f Pain Medicine and; g Gastrointestinal Medical Oncology, The University of Texas MD Anderson Cancer Center, Houston, TX, USA

**Keywords:** Laparotomy, Surgery, Recovery, Aspirin, Eicosapentaenoic acid

## Abstract

**Introduction:**

Major advances in therapies to optimize recovery after surgery have been limited by the lack of an animal model that can mimic major domains of postoperative sickness behavior in humans. We hypothesized that the integration of commonly impaired domains of quality of recovery in humans could be reproduced in a rat model.

**Objectives:**

To create a rat model that can mimic surgical recovery in humans.

**Methods:**

Adult male Sprague-Dawley rats were used in the development of a quality of recovery score after surgery. Six physiological parameters or behaviors were tested in naive, sham, and laparotomized animals. A quality of recovery score was constructed and ranged from 18 (no impairment) to 0 (gross impairment). We treated animals with a nutraceutical intervention consisting of aspirin and eicosapentaenoic acid. Inflammatory markers and specialized proresolving mediators were measured in serum and the intestinal mucosa of rats, respectively.

**Results:**

We observed a significant reduction in quality of recovery scores on postoperative days 1 (median, interquartile: 6 [4.75–8.25] vs naive rats: 17.5 [15.5–18]), 2 (median, interquartile: 13 [11.25–13.25], *P* < 0.001 vs naive rats: 17 [17–18], *P* = 0.001), and 3 (median, interquartile: 14.5 [13.5–16] vs naive rats: 17 [15.75–18], *P* < 0.02). Surgery promoted a significant increase in the concentrations of inflammatory cytokines, but it reduced levels of interleukin-12p70 and macrophage colony-stimulating factor. Lipoxin B4 and 13-HODE were significantly higher in laparotomized rats. Aspirin + eicosapentaenoic acid substantially improved recovery scores and modulated the postsurgical inflammatory response.

**Conclusion:**

Our novel rat model can be used to study mechanisms governing surgical recovery in rats.

## Abstract 2

Quantification of recovery after surgery can objectively perform in rats. Aspirin and eicosapentaenoic acid can modulate inflammation and enhance recovery after surgery.

## 1. Introduction

Each year, millions of patients undergo abdominal surgery worldwide.^33^ Despite significant clinical efforts, up to a half of those patients still report inadequate recovery after surgery, indicating the need for novel strategies to improve postoperative outcomes including functional recovery.^21^ Therefore, development of a preclinical model of recovery after laparotomy (open abdominal surgery) would be impactful and beneficial to patients, caregivers, and healthcare systems.^4,21^

The combined effects of tissue injury and anesthetics are responsible for a highly orchestrated release of proinflammatory and anti-inflammatory molecules (eg, cytokines and chemokines).^37^ Inflammatory mediators acting on the nervous system mediate symptoms of “sickness behavior” including pain-like behaviors, nausea and vomiting, anhedonia, lack of appetite, and fatigue.^8^ These symptoms are commonly seen after surgery, particularly after major operations.^35^ Unfortunately, anti-inflammatory drugs are mostly ineffective because of dose-limiting adverse events and the fact that although they may actually suppress inflammation, they do not actively promote its resolution.^25^

Resolution of inflammation is not a passive process but rather involves active anti-inflammatory mechanisms, for example, induction of specialized proresolving mediators (SPMs; lipoxins, resolvins, protectins, and maresins). Specialized proresolving mediators are reduced immediately after surgery.^5,34^ Low circulating concentrations of lipoxin A4 and resolving D1 were reported after major cancer hepatobiliary surgery.^5^ Thus, it can be theorized that the administration of donor molecules for SPMs would resolve inflammation and accelerate recovery of symptoms after surgery.

The primary aim of this study is to study the practicability of a quality of recovery score and its capacity to be modified by surgery and, secondarily, explore whether a nutraceutical intervention can positively impact postoperative recovery.^9,34^ We choose eicosapentaenoic acid (EPA) as it is a known donor of E-series resolving molecules (such as RvE). RvE1 has shown potent anti-inflammatory effects and also inhibits sensory disturbances in inflammatory pain models.^40^

## 2. Methods

### 2.1. Animals and treatments

Pathogen-free adult male Sprague-Dawley rats (8–10 weeks old; Envigo, Indianapolis, IN) were housed 2 to 3 per cage in a light- and temperature-controlled room (12:12-hour light–dark cycle, lights on at 7:00 am) during the acclimation period. During testing, rats were singly housed. Rats had food and water available ad libitum. All procedures were approved by the MD Anderson Cancer Center Institutional Animal Care and Use Committee.

### 2.2. Laparotomy surgery

Animals' weight ranged from 322 to 427 g before surgery. The rat laparotomy surgery was performed as previously described.^15,28^ Sham surgery consisted of exposure to isoflurane anesthesia alone and shaving of the abdominal skin. Laparotomized and sham animals were recovered for a minimum of 1 hour after surgery before return to the home cage. Naive animals were gently handled and tested during a period of 14 days in parallel with experimental rats.

To estimate adverse range of outcomes in physiological and behavioral parameters of recovery (for calculation of the percent maximum possible effect (see below)), we used a modified gut ischemia–reperfusion injury model.^26^ Briefly, after exposure of the abdominal cavity, the superior mesenteric artery and small bowel (1 cm) were occluded with atraumatic microvascular clamps for 20 minutes of ischemia, and then, the clamps were removed for reperfusion. Then, all animals were returned to their cages for observation.

### 2.3. Behavioral assessment of postoperative recovery

An animal model that resembles surgical recovery in humans is not existent. We selected and tested 6 different physiological or behavioral parameters known to be substantially impacted in the quality of recovery-15 questionnaire in humans undergoing abdominal surgery, including food consumption, social interaction, anhedonia (depression-like behaviors), ambulation (physical activity), and intestinal transit time.^21,35^

### 2.4. Food consumption, weight gain, and intestinal transit time

We quantified the amount of water (milliliter) and food (grams) that was ingested daily. The average intake of food and water over the 3 days before laparotomy was used as the baseline. Weight gain was calculated as the change (%) from baseline (preoperative) on days 1, 2, and 3 after surgery. Postoperative ileus delays surgical recovery in humans.^16^ In this work, the intestinal transit was measured in all animals and defined as the time (in minutes) from the end of a surgery or anesthesia (sham animals) to the first defecation after surgery. Naive animals had their intestinal transit time randomly measured in their cages during their daylight cycle.

### 2.5. Mechanical allodynia and locomotor activity

We consider allodynia at the incision site as one of our components of the surgical recovery model.^39^ Allodynia was assessed using a Von Frey test. The “up-down” Von Frey method used to determine the mechanical force required to elicit an abdominal response in 50% of animals.^11^ Reduced ambulation is considered a sign of slow recovery after surgery.^28^ The total distance (meters) in the horizontal direction was recorded during 12 hours (dark–light cycle).

### 2.6. Sucrose preference test and juvenile social exploration test

The sucrose preference test was used as an indicator of laparotomy-induced anhedonia.^18^ One bottle contained plain drinking water, and the second had sucrose (1%) solution. Sucrose preference was calculated as a percentage of the volume of sucrose intake over the total volume of fluid intake. Anxiety-like behaviors were tested in each experimental adult rat before (3 consecutive days) and after laparotomy (1, 2, 3, and 6) as previously described.^7,14^ Briefly, adult rats were placed in a new cage for 60 minutes before a juvenile stimulus rat (4–5 weeks old; 85–100 g) was added to the cage. After 3 minutes of exploration, each rat was returned to their home cage.^7^

### 2.7. Recovery score after surgery

Behavioral or physiological endpoints for each animal were calculated as % maximal possible effect (MPE) as follows: %MPE = (Test − baseline/maximum deficit − baseline) × 100, except for sucrose preference. The following maximum deficit values for each parameter were obtained from animals with intestinal ischemia: food consumption (0 mg), intestinal transit (720 minutes), sucrose preference (5%), mechanical allodynia (0.04 g), horizontal travelled distance (100 m), and juvenile social exploration (20 seconds). Preoperative tests in each animal served as baseline values to calculate the %MPE.

Each endpoint was assigned a score ranging from 3 to 0, based on %MPE: 3 = 100% to 75%; 2 = 50% to 74%; 1 = 25% to 49%; or 0 = 0% to 24%. The scores from each day were summed for a maximum of 18 (indicating little to no impairment) or a minimum of 0 (indicating gross impairment).

### 2.8. Eicosapentaenoic acid and aspirin treatment

**Figure 1.**
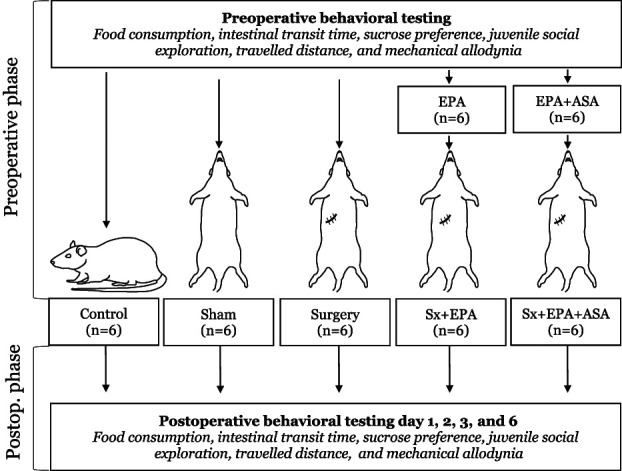
The figure illustrates the experimental design used in our study. ASA, aspirin; EPA, eicosapentaenoic acid; Postop, postoperative; Sham, animals received isoflurane anesthesia alone; Sx, surgery.

Aspirin is a known anti-inflammatory drug, but it also promotes the release of aspirin-triggered resolvin D1 (AT-RvD1), a D-series resolving as the results of acetylation of the cyclooxygenase-2 enzyme.^36^ Eicosapentaenoic acid is a known donor of E-series resolving molecules.^31^ Aspirin or the combination of aspirin + EPA were given to animals (n = 6/group) from day −7 of surgery to postoperative day 6 to investigate their effects on recovery after surgery, cytokines and SPMs (Fig. 1). Aspirin (10 mg/kg/d) was diluted in tap water and administered orally. Eicosapentaenoic acid ethyl ester was added at a dietary concentration of 1% by weight (10 g/kg diet). Animals consumed EPA 1% (Research Diets, Inc., New Brunswick, NJ) pellets ad libitum as part of their daily food intake. The control diet contained 320 (g/kg diet) sucrose, 200 casein, 220 corn starch, 3 dl-methionine, 35 AIN 76 salt mix, 10 AIN 76 mineral mix, 2 choline chloride, 60 fiber (cellulose), and 150 corn oil.^12,32^ The EPA diet was the same as the control diet except that corn oil is partially replaced with EPA ethyl ester at 1% of the total diet weight to provide the same number of calories as the corn oil that was replaced.^10^ Animals receiving any nutraceutical treatment were randomly allocated to each treatment and started experiments after the initial cohort of rats for establishing our model was developed.

### 2.9. Cytokine analysis

We collected serum from euthanized animals on day 0 (preoperative) and days 1 and 7 after sham surgery or laparotomy. Naive animals served to compare the effect of isoflurane and surgery on the different analytes. Systemic levels of cytokines, chemokines, and growth factors from serum were determined by using the Bio-Plex Pro Rat Cytokine 23-Plex magnetic bead-based multiplex immunoassay (Bio-Rad, #12005641, Hercules, CA) per manufacturer recommendations. Samples were processed by an investigator blinded to animals' allocation treatment.

### 2.10. Lipid analysis

The gut can initiate a complex and orchestrated local and, subsequently, systemic inflammatory reaction in response to intestinal manipulation.^3^ Animals were euthanized on day 0 (preoperative) and days 1 and 7 after sham surgery or laparotomy. The gut mucosa was collected and snap frozen in liquid nitrogen and stored at −80 ^o^C.

Column temperature was set at 30°C. Mobile phase A was 0.1% formic acid (Sigma-Aldrich, St. Louis, MO) in water, and mobile phase B was 0.1% formic acid in acetonitrile (Fisher Scientific, Pittsburgh, PA). The gradient conditions were as follows: the initial mobile phase was made up of 80% of mobile phase A and 20% of mobile phase B and hold for 1 minute. Mobile phase B was linearly ramped to 40% at 4 minutes and hold until 12.5 minutes. The gradient was changed to 90% of B at 21 minutes and hold until 23 minutes. Mobile phase B was further increased to 98% at 24 minutes and hold until 28 minutes. At 28.1 minutes, the gradient was set back to 20% of B and hold until 33 minutes. The flow rate was 0.4 mL/minute and total run time was 33 minutes. Samples were processed by an investigator blinded to animals' allocation treatment.

### 2.11. Data analysis

Assuming a mean score of 17 (SD: 4 points), we estimated that a total of 12 animals (n = 6 per group) would be needed to show a 50% reduction in the quality of recovery score in operated animals compared with naive rats (1 − β = 0.8).

Data are shown in mean ± SEM or median with interquartile range. One- or two-way repeated measures analysis of variance (time × treatment) was used to analyze and compare the effect of surgery and each intervention on animals' behaviors and quality of recovery. The analysis was followed by post hoc Dunnett tests to determine the statistical significances, when indicated. Kruskal–Wallis tests were used to compare nonparametric variables. To further distinguish how recovery occurred between each treatment group, we estimated their area under the curve (AUC) and compared them with one-way analysis of variance. An adjusted *P* < 0.05 was considered statistically significant.

## 3. Results

### 3.1. Effect of surgery and isoflurane anesthesia on parameters of recovery

**Figure 2.**
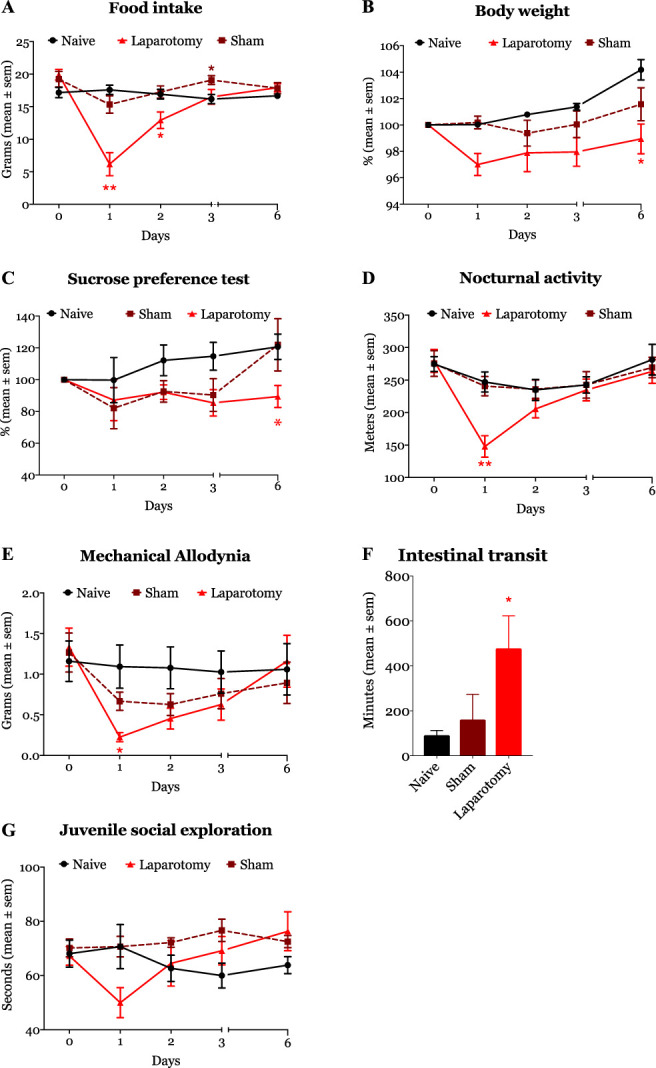
Laparotomy surgery (solid red line/object) significantly impacted 5 of the 6 study tested domains. Food intake, sucrose preference, nocturnal activity, and mechanical nociceptive thresholds were significantly reduced after surgery. Surgery also significantly increased intestinal transient. *P < 0.05, **P < 0.01, and ***P < 0.001.

A total of 48 rats were included in the study. Two animals died during surgery and were replaced accordingly. Laparotomized animals (n = 6) showed a substantial reduction in food intake compared with naive rats (n = 6) (Fig. 2A). The difference in food consumption reached statistical significance on days 1 (naive: 17.25 [16.25–19] vs laparotomy: 6.75 [2–8.87], *P* = 0.001) and 2 after surgery (naive: 17 [15.25–18.63] vs laparotomy: 12.5 [9.87–16], *P* = 0.001, Fig. 2). As shown in Figure 2B, the impact of reduced food intake on weight gain after surgery was only significant on postoperative day 6 (naive: 5% [1.9–6.5] vs laparotomy: 0.1% [−3.98–0.7], *P* = 0.03). Surgery also significantly impacted sucrose preference. Although we observed a reduction in preference towards sucrose in operated animals in comparison with naive rats on day 6 after surgery, the difference was not statistically significant (Fig. 2C).

Laparotomy reduced the locomotor activity (travelled distanced in meters) of animals (Fig. 2D). On postoperative day 1, operated animals had a substantial reduction in the horizontal travel distance (271.20 [248.70–302.01] m) compared with naive animals (138.92 [117.40–172.61] m, *P* = 0.016). In terms of nociception, laparotomized animals showed significant mechanical allodynia on day 1 (0.28 [0.13–0.55] grams, *P* < 0.037) in comparison with naive rats (1.2 [0.49–2.55] grams). Although operated animals remained allodynic on postoperative days 2 and 3, the difference was not statistically significant (Fig. 2E). Surgery delayed the intestinal transit times, as evidenced by a longer transit time in laparotomized animals (617 [42.5–30,201] minutes) than naive rats (72.5 [63–113.3] minutes, *P* = 0.043, Fig. 2F). Finally, although social exploration was slightly impaired on day 1 after laparotomy, the overall difference with naive animals did not reach statistical significance (Fig. 2G).

General anesthesia (sham animals, n = 6) did not significantly affect animals' weight (data not shown). In addition, compared with naive animals, isoflurane anesthesia did not cause any statistically significant change in food consumption, sucrose preference, intestinal transit time, abdominal allodynia, social exploration, and locomotor activity (data not shown).

### 3.2. Effect of surgery and isoflurane anesthesia on recovery scores

**Figure 3.**
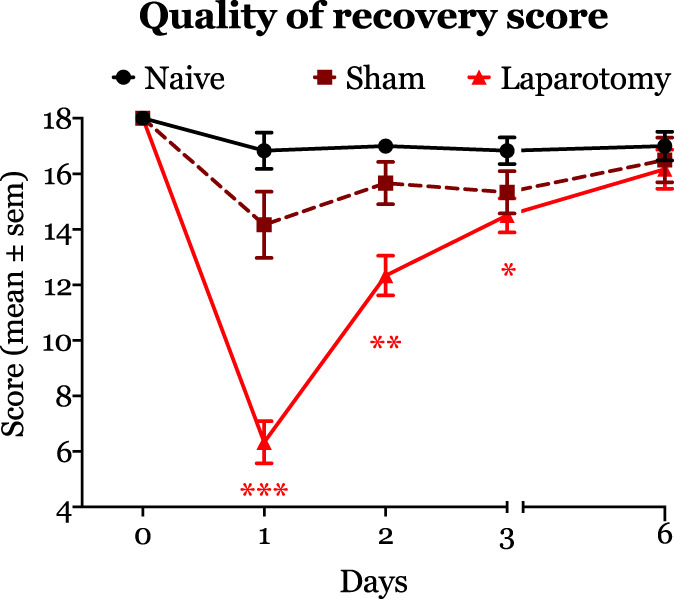
The figure depicts recovery after surgery as measured by our novel quality of recovery score. Laparotomy surgery caused a significant reduction in quality of recovery scores on postoperative days 1 (median, interquartile: 6 [4.75–8.25] vs naive rats: 17.5 [15.5–18]), 2 (median, interquartile: 13 [11.25–13.25], *P* < 0.001 vs naive rats: 17 [17–18], *P* = 0.001), and 3 (median, interquartile: 14.5 [13.5–16] vs naive rats: 17 [15.75–18], *P* < 0.02). *P < 0.05, **P < 0.01, and ***P < 0.001.

Collectively, the abnormal behaviors observed in laparotomized animals reduced the quality of recovery score compared with sham and naive animals (Fig. 3), and the trajectory of the curve mimicked a previous report in humans.^21^ The median (interquartile range) recovery scores in laparotomized animals were statistically significantly lower than naive rats on days 1 (naive: 17.5 [15.5–18] vs laparotomy: 6 [4.75–8.25], *P* = 0.144), 2 (naive rats: 17 [16.75–17.25] vs laparotomy: 13 [11.25–13.25], *P* = 0.001), and 3 after surgery (naive rats: 17 [15.75–18] vs laparotomy: 14.5 [13.5–16], *P* = 0.029, Fig. 3). Further analysis of the AUC indicated that surgery caused a significant reduction in recovery (total area ± SE: 80.92 ± 3.96) compared with naive animals (102 ± 2.91, *P* < 0.003), but this effect was not seen in sham rats (94.25 ± 4.84, *P* = 0.376) in comparison with naive rats.

Animals that had only isoflurane-maintained general anesthesia showed a slight decrease in the recovery scores on postoperative day 1 compared with naive rats. However, the overall change in the score was not statistically significantly different compared with naive animals.

### 3.3. Effect of surgery and isoflurane anesthesia on inflammatory mediators and specialized proresolving mediators

On postoperative day 1, we observed that, compared with naive animals, surgery promoted a significant increase in the concentrations of interleukin-1a (IL-1a), IL-6, IL-10, and interferon (IFN) gamma (Table 1). By contrast, the serum levels of IL-12p70 and macrophage colony-stimulating factor (M-CSF) were significantly lower in operated animals than naive rats also on postoperative day 1. Seven days after surgery, the circulating levels of IL-1a, IL-1β, IL-5, IL-7, tumor necrosis factor, and IFN-γ remained significantly higher in operated rats than in nonoperated naive animals. As shown in Table 1, isoflurane anesthesia did not significantly impact the serum concentrations of the analytes.

**Table 1:** Effect of isoflurane anesthesia, surgery, and their combination on inflammatory markers.

Cytokine	Naive rats (n = 6)	Sham rats (isoflurane anesthesia alone)		Laparotomy rats		*P*
		Postoperative day 1 (n = 6)	Postoperative day 7 (n = 6)	Postoperative day 1 (n = 6)	Postoperative day 7 (n = 6)	
IL-1a	62.47 (1.75–210.7)	129.6 (50.52–297.9)	69.46 (5.77–269.4)	400.3 (235.3–520.6)*	371.5 (173.9–611.4)†	0.001
IL-1β	282.8 (8.24–586.5)	152.8 (16.23–561)	329 (18.39–1073)	926.8 (414.1–1510)	1013 (542.9–1466)†	0.005
IL-2	700.7 (396.5–2174)	467.6 (55.11–3092)	1246 (330.3–3434)	1335 (170.8–1979)	939 (280.9–6052)	0.688
IL-4	862.8 (426.6–1248)	413.9 (43.23–868.8)	999.6 (19.02–1099)	433.5 (209.3–719)	667.8 (118.2–912.1)	0.376
IL-5	516.8 (67.19–587.2)	398.7 (64.61–534.4)	185.7 (89–491.9)	429.9 (265.3–569.5)	561 (483.3–673)†	0.104
IL-6	663.2 (249.7–5090)	1197 (307.6–5071)	1121 (83.34–3440)	6382 (4832–8780)†	7210 (3718–7081)	0.009
IL-7	20.34 (2.25–266.7)	35.04 (2.88–151.8)	196.6 (4.26–341.4)	88.27 (22–210.1)	260.5 (172.7–392.7)†	0.139
IL-10	4.26 (1.73–15.93)	30.12 (3.21–175.6)	54.37 (5.37–236.8)	128.7 (15.78–292.5)†	126.5 (5.48–261.4)	0.002
IL-12p70	1128 (752.7–1468)	1019 (195–1306)	1372 (14.55–1565)	239.9 (61.9–461.7)†	818.5 (308.5–1190)	0.192
IL-13	267.1 (36.19–502.4)	305.1 (224.9–435.9)	169.8 (34.49–621.4)	305.1 (150.6–578.8)	483.7 (331.4–869.5)	0.312
IL-17A	64.39 (14.23–140.9)	77.18 (3.53–121.4)	113 (3.03–166.7)	77.7 (17.1–147.5)	103.3 (19.52–140.3)	0.794
IL-18	2511 (1293–3907)	2053 (1134–4044)	3482 (196.5–4973)	4056 (2134–6036)	5805 (3359–7091)†	0.02
TNFα	75.41 (45.52–652.7)	200.41 (44.5–1140)	826 (59.84–1294)	810 (413–1763)	753.7 (538.5–2211)†	0.01
GM-CSF	47.27 (1.53–187.5)	29.06 (2.13–112.1)	162.2 (2.58–207.2)	184.4 (113.8–271.3)	185.5 (18.01–272.4)	0.133
IFNγ	110.2 (23.08–1022)	198.6 (26.8–895.3)	48.04 (4.96–1281)	1217 (906.3–1738)†	1327 (1001–1802)*	0.005
MCP1	326.4 (129–648.9)	321.5 (215.3–576.4)	532.8 (226.5–736.1)	338.2 (261.8–432.5)	433.2 (294–788.3)	0.944
M-CSF	106.4 (71.45–165.9)	88.2 (48.4–138.9)	141 (56.8–178.1)	20.61 (7.4–30.38)*	65.2 (19.16–95.46)	0.02
MIP-1a	50.32 (32.56–71.46)	29 (4.83–53.13)	80.2 (2.15–97.04)	41.99 (20.52–56.8)	52.7 (30.57–69.28)	0.875
MIP-3a	28.37 (18.53–50.89)	23.94 (14.4–38.6)	35.1 (3.4–31.1)	21.07 (9.4–31.19)	9.1 (3.4–28.2)	0.597
G-CSF	50.17 (0.41–65.84)	42.88 (9.54–55.87)	52.75 (3.33–79.01)	34.06 (15.14–65.92)	44.75 (18.6–89.62)	0.97
RANTES	36.6 (22.42–148.5)	71.53 (26.31–195.2)	66.55 (40.88–162)	72.54 (51.51–297.2)	110.3 (44.51–158.4)	0.455
GRO-KC	60.92 (29.63–85.05)	39.83 (16.4–72.76)	55.84 (4.77–85.21)	32.2 (15.88–97.92)	57.08 (40.19–101.2)	0.847
VEGF	221.2 (127.5–363.3)	257.5 (210.8–298.4)	268.8 (9.02–414.7)	150.7 (64.66–309.1)	295.3 (191.5–485.8)	0.915

* *P* < 0.01.

† *P* < 0.05.

G-CSF, granulocyte colony-stimulating factor; GM-CSF, granulocyte–monocyte colony-stimulating factor; GRO-KC, growth-regulated oncogene-keratinocyte chemoattractant; IFNγ, interferon gamma; IL, interleukin; MCP1, monocyte chemoattractant protein 1; M-CSF, macrophage colony-stimulating factor; MIP-1a, macrophage inflammatory protein 1a; MIP-3a, macrophage inflammatory protein 3a; RANTES, Regulated upon Activation, Normal T Cell Expressed and Presumably Secreted; TNFα, tumor necrosis factor alpha; VEGF, vascular endothelial growth factor.

Dunnett's correction between naive rats and laparotomy animals on days 1 and 7 after surgery.

We measured the concentrations of prostaglandins E2 and E3 in the intestinal mucosa of naive, sham, and laparotomized animals. We observed that the concentrations of lipoxin B4 and 13-HODE were significantly higher in laparotomized animals than in naive rats. Neither isoflurane anesthesia nor surgery impacted the intestinal concentrations of prostaglandins E2 and E3 in comparison with naive animals (Table 2). Interestingly, isoflurane anesthesia (sham rats) significantly raised the intestinal levels of resolvin D1 but not those of the other SPMs that we assayed (Table 2).

**Table 2:** Effect of anesthesia, surgery, and aspirin plus eicosapentaenoic acid on prostaglandins (E2 and E3), lipoxin B4, and resolvins (E-series and D-series).

Metabolite, ng/mg of protein	Naive rats (n = 3)	Laparotomy rats		*P*^1^	Laparotomy + ASA + EPA rats		*P*^2^	Naive rats ASA + EPA day 7 (n = 4)	*P*^3^
		Postoperative day 1 (n = 4)	Postoperative day 7 (n = 4)		Postoperative day 1 (n = 4)	Postoperative day 7 (n = 4)			
PGE2	7.92 (4.5–24.63)	16.6* (2.13–27.22)	2.67 (2.13–13.37)	0.002	2 (0.73–4.43)†	1.14 (0.64–2.21)	0.03	1.57 (0.65–1.69)	0.228
PGE3	0 (0–0)	0 (0–0)	0 (0–0)	0.999	0.03 (0–0.05)†	0 (0–0)	0.08	03 (0–0.21)	0.428
LXB4	0.49 (0.33–0.96)	2.35 (0.76–3.55)*	0.15 (0–0.57)	0.087	0.1 (0–0.55)‡	0.06 (0–0.21)	0.037	0.2 (0–0.83)	0.542
13-HODE	10.17 (4.15–29.83)	22 (16.95–51.17)*	11.05 (3.91–40.38)	0.062	10.58 (6.22–26.8)	4.22 (3.52–4.6)	0.072	5.48 (3.62–6.67)	0.185
5-HETE	1.35 (0.18–2.37)	0.72 (0.51–1.39)	0.71 (0.2–1.7)	0.362	0.33 (0.26–1.08)	0.23 (0.17–0.72)	0.1	0.27 (0.17–0.39)	0.4
12-HETE	0.6 (0.16–1.65)	0.62 (0.45–0.77)	0.47 (0.14–1.72)	0.165	0.23 (0.12–0.35)	0.28 (0.12–0.35)	0.055	0.16 (0.08–0.25)	0.228
15-HETE	2.73 (1.07–8.17)	4.16 (2.15–6.82)	1.39 (0.55–6.49)	0.08	1.5 (0.73–1.93)	0.85 (0.51–1.15)	0.074	0.55 (0.37–1.61)	0.114
18-HETE	0.37 (0.2–0.59)	0.86 (0.45–2.27)	0.89 (0.47–1.37)	0.586	1.29 (0.56–10.14)	1.16 (0.5–1.64)	0.316	5.46 (0.94–10.52)	0.05
17-HDHA	1.93 (0.38–3.55)	0.51 (0.19–2.32)	0.94 (0.29–3.05)	0.63	0.24 (0.18–2.78)	0.62 (0.22–0.68)	0.155	3.93 (1.07–5.08)	0.4
RvD1	0.06 (0–0.07)	0.07 (0.01–0.12)	0.21 (0.9–0.36)	0.017	0.09 (0.08–1.54)	0.11 (0.08–0.13)	0.456	0.06 (0–0.13)	0.714
RvD3	0.02 (0.01–0.03)	0.03 (0.02–0.04)	0.06 (0.03–0.15)	0.303	0.03 (0.02–0.36)	0.03 (0–0.04)	0.612	0.06 (0–0.13)	0.857
RvD4	0.25 (0.16–0.29)	0.3 (0.14–0.44)	0.27 (0.16–0.37)	0.633	0.18 (0.15–2.29)	0.23 (0.17–0.29)	0.458	0.12 (0–0.25)	0.424
RvD5	0 (0–0.5)	0 (0–0)	0 (0–0)	0.186	0.06 (0.01–1.24)	0 (0–0)	0.311	0 (0–0)	0.542
RvE1	0.02 (0–0.04)	0.05 (0.04–0.13)	0.31 (0.07–0.57)	0.319	0.06 (0.02–4.25)	0.03 (0.01–0.16)	0.516	0.79 (0.24–2.19)	0.05

*P* value^1,3^ for Kruskal–Wallis analysis. Symbols indicate differences after multiple comparisons (Dunn's test). *P* value^2^ comparing laparotomy vs laparotomy + ASA + EPA. * †*P* < 0.05 * * † †*P* < 0.01. RvD2 levels are not shown because they are undetectable.

EPA, eicosapentaenoic acid; HETE, Hydroxyeicosatetraenoic acid; HDHA, hydroxydocosahexaenoic acid; HODE, Hydroxyoctadecadienoic acid; LXB4, Leucotriene B4; PG, Prostaglandin; Rv, resolvin.

### 3.4. Impact of aspirin and aspirin + eicosapentaenoic acid on individual parameters of recovery

Because aspirin is an anti-inflammatory drug and can trigger resolving D1 production, and EPA can serve as a donor of SPMs, we investigated whether aspirin alone or the combination of aspirin + EPA impacted animals' weight, sickness behaviors, and recovery after surgery.

**Figure 4.**
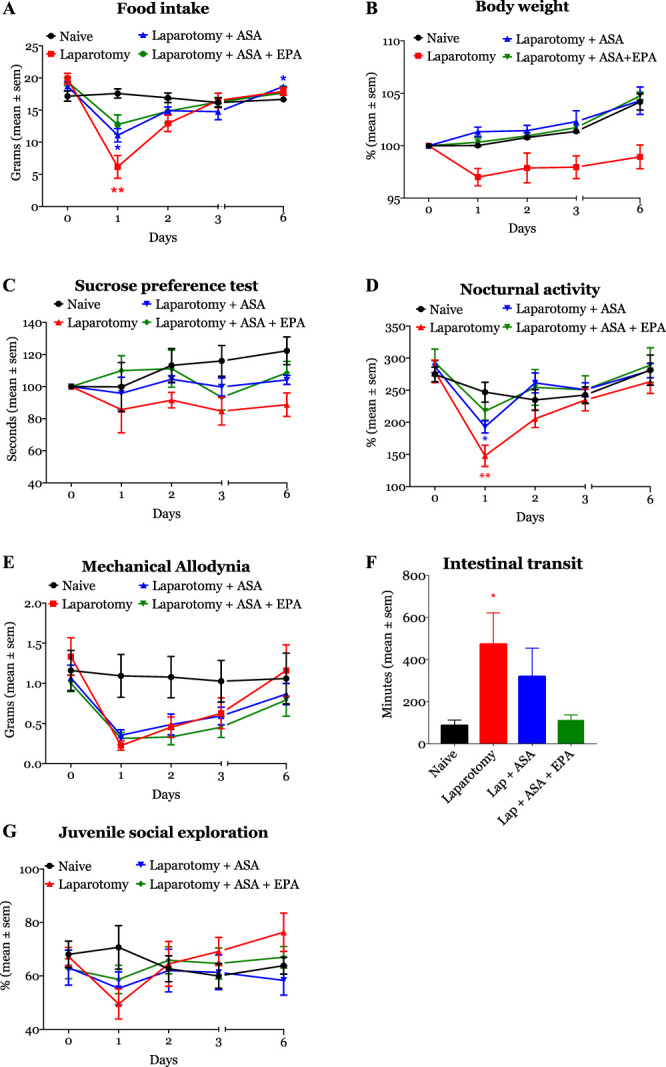
Aspirin (ASA) and EPA are modulators of inflammation. Animals treated with the combination of ASA + EPA (green line/object) showed a significant improvement in 4 of the 6 behavioral domains tested. ASA alone (blue line/object) has a small or modest effect on parameters of recovery. *P < 0.05, **P < 0.01, and ***P < 0.001. EPA, eicosapentaenoic acid.

As shown in Figure 4A, food consumption was significantly lower in operated rats treated with aspirin (11 [9.25–13.63] grams, *P* = 0.001) than in naive animals (17.25 [16.25–19] grams) on postoperative day 1. From day 2 after surgery to the end of the experiment, food consumption was comparable between the laparotomized animals treated with aspirin and aspirin + EPA and naive rats (Fig. 4A). Aspirin and aspirin + EPA prevented weight loss in shams and operated animals (Fig. 4B). Contrarily, aspirin + EPA (15.25 [8.62–16.63]) attenuated the impact of surgery on food consumption (naive rats: 17.25 [16.25–19] grams, *P* = 0.06). Aspirin and aspirin + EPA did not affect sucrose consumption (Fig. 4C).

Our data also showed that laparotomized animals treated with aspirin (193.85 [168.09–214.46] m) had a significant reduction in the traveled distance on the first postoperative night compared with naive rats (240.31 [218.98–288.23] m, *P* = 0.017, *P* = 0.04, Fig. 4D). Contrarily, on day 1, horizontal activity was not statistically significantly reduced among rats treated with aspirin + EPA (221.81 [192.07–246.03] m), compared with naive animals (240.31 [218.98–288.23] m, *P* = 0.388). On the following postoperative nights, the locomotor activity of the animals was similar across all experimental groups in relationship to naive rats (Fig. 4D). There were no differences in mechanical allodynia after surgery between different groups of treatment (Fig. 4E). Aspirin and aspirin + EPA treatment also restored intestinal transit time (Fig. 4F); laparotomy rats treated with aspirin (191 [70–720] minutes) or aspirin + EPA (112.5 [61.25–210]) had similar transit times to naive animals (72.5 [63–113.3] minutes, *P* = 0.252 and *P* = 0.997). Social exploration was not impacted by the administration of aspirin and aspirin + EPA (Fig. 4G).

Aspirin + EPA treatment impacted food and weight in shams compared with naive animals. The difference in body weight reached statistical significance on day 3 (*P* = 0.028). Similarly, food consumption was larger in aspirin + EPA sham animals than naives (*P* = 0.006). Neither aspirin nor aspirin + EPA altered sucrose preference, social interaction, mechanical allodynia, locomotor activity, or intestinal transit in sham animals compared with naive rats (data not shown).

### 3.5. Effect of aspirin and aspirin + eicosapentaenoic acid anesthesia on quality of recovery

**Figure 5.**
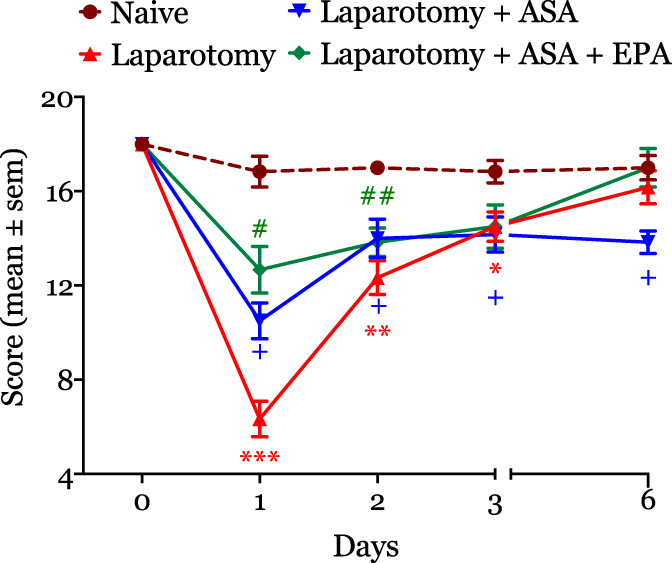
The figure illustrates the effect of ASA and ASA + EPA on recovery after surgery. ASA + EPA caused a significant improvement in the quality of recovery score that was evident on postoperative day 1 (median, interquartile: 6 [4.75–8.25] vs ASA + EPA rats: 12 [10.75–15.25]). By contrast, ASA treatment worsened recovery. *P < 0.05, **P < 0.01, and ***P < 0.001. #P, 0.05, ##P, 0.01, and ###P, 0.001. ASA, aspirin; EPA, eicosapentaenoic acid.

We observed that operated animals receiving treatment with aspirin + EPA had an accelerated recovery that was evident on days 1 and 2 after surgery (Fig. 5). The difference in AUCs was not statistically significantly different between aspirin + EPA (90 ± 5.07) and naive rats (102 ± 2.91, *P* = 0.144), suggesting a protective effect of aspirin + EPA on surgical recovery. On the other hand, although those treated with aspirin had an initial improvement in recovery (postoperative days 1 and 2), the effect disappeared on days 3 and 6. The AUC analysis indicated that aspirin did not have any significant effect on recovery (total area ± SE: 82.58 ± 3.9) compared with naive rats (102 ± 2.01, *P* < 0.008). These findings suggest that aspirin caused a late impairment in the quality of recovery. We did not observe any impact of aspirin or aspirin + EPA on sham animals (data not shown).

### 3.6. Effect of aspirin and aspirin + eicosapentaenoic acid on inflammation and specialized proresolving mediators

As shown in Table 3, neither aspirin nor aspirin + EPA significantly affected the serum concentrations of the studied cytokines on day 1 after surgery. However, we observed that on postoperative day 7, operated animals treated with aspirin had significantly higher median levels of IL-2 and M-CSF compared with nontreated operated animals. Treatment with aspirin + EPA substantially reduced concentrations of IL-1α, IL-5, IL-7, IL-18, and IFN-γ in operated animals compared with the laparotomized nontreated rats.

**Table 3:** Effect of aspirin and eicosapentaenoic acid on inflammatory markers after laparotomy.

Cytokine, pg/mL	Postoperative day 1 (median, IQR)			Postoperative day 7 (median, IQR)			*P*
	Laparotomy (n = 6)	Lap + ASA (n = 6)	Lap + ASA + EPA (n = 6)	Laparotomy (n = 6)	Lap + ASA (n = 6)	Lap + ASA + EPA (n = 6)	
IL-1α	400.3 (235.3–520.6)	135.1 (58.24–397)	333.1 (59.8–400.9)	371.5 (173.9–611.4)	348.8 (119.8–373)	46.4 (6.49–164.3)*	0.011
IL-1β	926.8 (414.1–1510)	290 (106.5–1299)	914.6 (178.2–1164)	1013 (542.9–1466)	637.1 (21.81–1049)	210 (41.63–624.3)	0.123
IL-2	1335 (170.8–1979)	1956 (994.9–3435)	4639 (1427–4843)	939.6 (290.8–6052)	4080 (2579–6827)*	2991 (1588–4885)	0.06
IL-4	433.5 (209.3–719)	335.7 (174.4–707.4)	900 (171.7–1051)	667.8 (118.2–912.1)	335.7 (174.4–707.4)	892.6 (501.6–1141)	0.375
IL-5	429.9 (265.3–569.5)	361. (233.8–550.4)	608.6 (193.6–671.4)	561 (483.3–673)	579 (527.1–609.1)	309.8 (118.4–409)*	0.02
IL-6	6382 (4832–8780)	3512 (2002–5268)	6708 (2077–6769)	6382 (4832–8780)	6382 (3718–7081)	3551 (249.7–7057)*	0.08
IL-7	88.27 (22–210.1)	78.33 (26.61–184.1)	104 (104–303.2)	260.5 (172.2–392.7)	271.2 (202.2–330.7)	34.34 (3.62–285.6)*	0.602
IL-10	128.7 (15.78–292.5)	124.6 (44.50–257.1)	301.7 (169.2–350.3)	126.5 (5.48–261.4)	266.9 (152.6–305.4)	286.2 (184.7–336.9)	0.084
IL-12p70	239.9 (61.9–461.7)	476.3 (169.2–1032)	1414 (230.6–1574)	818.5 (308.5–1190)	1225 (733.4–1359)	1383 (617.5–1676)	0.121
IL-13	305.1 (150.6–578.8)	290.7 (149.8–486.5)	613.5 (146.9–767)	483.7 (331.4–869.5)	650.8 (470.2–814.2)	483 (170.4–797.7)	0.982
IL-17A	77.7 (17.1–147.5)	70.8 (45.17–149.6)	151.7 (36.77–172.6)	103.3 (19.52–140.3)	151.8 (123.8–164.1)	84.84 (24.8–165.6)	0.734
IL-18	4056 (2134–6036)	1956 (994.9–3435)	4369 (1427–4843)	5805 (3359–7091)	4423 (3764–5210)	2405 (243.7–3904)†	0.08
TNFα	810 (413–1763)	493 (323.5–743)	1112 (523.3–1312)	753.7 (538.5–2211)	1059 (936.9–1386)	377 (107.8–744.3)	0.866
G-CSF	34.06 (15.14–65.92)	31.84 (10.63–53.6)	67.25 (14.9–83.86)	44.75 (18.6–89.62)	67.71 (20.59–79.84)	59.64 (36.07–73.55)	0.81
GM-CSF	184.4 (113.8–271.3)	64.75 (26.17–142.8)	187. (92.19–228.8)	185.5 (18.01–272.4)	162.8 (117.5–233.9)	129.5 (21.11–286)	0.802
IFNγ	1217 (906.3–1738)	576 (301.1–1028)	1297 (306.1–1494)	1327 (1001–1802)	1359 (1064–1517)	181.1 (21.36–955.8)†	0.05
MCP1	338.2 (261.8–432.5)	233.4 (108.9–797.1)	262 (101.2–797.1)	433.2 (294–788.3)	653.8 (468.6–749.4)	250.4 (123.7–803.5)	0.793
M-CSF	20.61 (7.4–30.38)	33.4 (14.34–67.06)	127.2 (19.47–151.8)	65.26 (19.16–95.46)	113.6 (75.2–132.9)*	94.08 (43.1–141)	0.08
MIP-1a	41.99 (20.52–56.8)	73.84 (51.02–97.5)	67.77 (11.88–87.02)	52.7 (30.57–69.28)	52.7 (30.57–97.5)	61.8 (24–86.74)	0.76
MIP-3a	21.07 (9.4–31.19)	15.8 (7.7–54.2)	14.5 (8.7–51.6)	9.1 (3.4–28.2)	44.03 (28.4–53)	44.9 (26.7–53.40)	0.135
RANTES	72.54 (51.51–297.2)	79.34 (40.97–208.3)	161.9 (49.94–203.1)	110.3 (44.51–158.4)	180.8 (76.95–213.6)	87.36 (6.67–253.9)	0.995
GRO-KC	32.2 (15.88–97.92)	26.81 (13.33–87.37)	67.88 (19.98–91.42)	57.08 (40.19–101.2)	74.95 (51.93–106.4)	41.9 (4.03–94.17)	0.917
VEGF	150.7 (64.66–309.1)	116.2 (50.22–319.2)	307.7 (70.42–292.9)	295.3 (191.5–485.8)	391.3 (244.7–455.2)	156.8 (3.73–380.3)	0.594

ASA, aspirin; EPA, eicosapentaenoic acid; G-CSF, granulocyte colony-stimulating factor; GM-CSF, granulocyte–monocyte CSF; GRO-KC, growth-regulated oncogene-keratinocyte chemoattractant; IFN, interferon gamma; IL, interleukin; IQR, interquartile range; MCP1, monocyte chemoattractant protein 1; M-CSF, macrophage CSF; MIP-1a, macrophage inflammatory protein 1a; MIP-3a, macrophage inflammatory protein 3a; RANTES, Regulated upon Activation, Normal T Cell Expressed and Presumably Secreted; TNFα, tumor necrosis factor alpha; VEGF, vascular endothelial growth factor.

* *P* < 0.05 vs laparotomy group.

† *P* < 0.01 vs laparotomy group.

The combination of aspirin + EPA did not have any significant effect on the concentrations of resolvins of the D and E series in the intestinal mucosa of operated rats compared with untreated laparotomized rats (Table 2). By contrast, the levels of PGE 2 and LXB4 were significantly lower in treated animals on day 1 after surgery compared with nontreated laparotomized animals. Aspirin + EPA significantly increased the concentrations of PGE3 after surgery. Only resolvin E1 was marginally increased in sham animals treated aspirin + EPA animals (Table 2).

## 4. Discussion

This study demonstrated that a rat model of recovery after surgery was successfully established. Furthermore, we found that the trajectory of postoperative recovery in rats is similar to that described in patients undergoing major surgery.^21^ Also, our series of experiments confirm and extend existing knowledge by demonstrating that in animals, laparotomy induces measurable parameters of sickness behavior.^19,28^ Laparotomized rats also showed abdominal mechanical allodynia as previously described by Charlet et al. However, the duration of allodynia was shorter in our study. The difference in the duration of the allodynia could be explained by differences in the models such as the implantation of electrodes and a transmitter for heart rate monitoring in the study by Charlet.^6^

Mood disorders including depression have been reported after surgery and can interfere with patients' recovery.^13,28^ We found that operated rats had a lower but not statistically significant preference to drink sweetened water. Thus, we can speculate that although bowel manipulation during surgery can cause bacterial translocation (immune challenge), the endotoxin levels in plasma are not high on day 1 after surgery to produce a significant rise in cytokines to active centers in the brain responsible for anhedonia.^2^ On the other hand, a late and sustained elevation in proinflammatory cytokines as we observed with cytokines such as IL-1β and IL-6 correlates with the late onset of anhedonia on day 6 after surgery.

Our study also supports previous research demonstrating that surgery is associated with an increased inflammatory response.^22^ We observed that the levels of most inflammatory cytokines were higher on day 6 than day 1 postoperatively. Although a late peak in cytokines is certainly possible, we cannot rule out that we might have missed the peak levels of some cytokines, which might have occurred on postoperative day 2 or 3. We also observed that IL-7 was elevated in operated rats. We speculate that intestinal manipulation might have been promoted the production and release of IL-7 from intestinal epithelial cells into the circulation.^38^

Macrophage colony-stimulating factor–treated monocytes express a substantial part of the M2 transcriptome.^29^ It is conceivable that low levels of M-CSF early after surgery could represent a mechanism to facilitate the production of M1- over M2-type macrophages and thus favor the secretion of proinflammatory cytokines such as tumor necrosis factor.^23^ Our experiments also demonstrated that the serum levels of IL12-p70 were diminished on day 1 after surgery. In patients with postoperative infections, IL-12p70 is elevated in comparison with patients with no infectious complications.^42^ In our experimental conditions, low circulating concentrations of IL-12p70 coincided with peak levels of IL-10, a known regulator of proinflammatory cytokines.^27^ Thus, the high measured levels of IL-10 in serum on day 1 after laparotomy were in response to surgical trauma and gut manipulation with the goal of preventing an exaggerated inflammatory response.^24^

This study shows the combination of aspirin + EPA improved after laparotomy recovery as measured by our novel recovery score. We found that the serum levels of IL-1α, IL-5, IL-6, IL-7, IL-18, and IFN-γ were lower after treatment with aspirin + EPA. We believe that the observed anti-inflammatory effects were predominantly driven by the combination of aspirin and EPA more than aspirin alone because each intervention impacts different groups of cytokines.^20,30^ We also observed that aspirin + EPA modulated the gut mucosa concentrations of PGE2, PGE3, and LXB4. A reduction in the PGE2 level is consistent with the combined effects of aspirin and EPA.^41^ However, we also observed an increase in PGE3. High levels of PGE3 in the gut of the animals could be the result of EPA treatment and as a negative feedback mechanism to decrease cyclooxygenase-2 induction and PGE2 synthesis.^1,41^ Although we did not observe any effect of aspirin + EPA treatment on E-series or D-series resolvins, naive animals fed with aspirin + EPA did show higher levels of RvE suggesting that surgery and/or bowel manipulation can interfere with RvE production.^34^ Finally, it is worth noticing that among all the measured parameters of sickness behavior, rats treated with the combination of aspirin and EPA showed a lesser impact of surgery on locomotor activity than those taking only aspirin.

It is also worth considering that our model to test recovery after surgery has limitations. First, it was developed for abdominal surgery. Hence, there might be surgery-specific differences in the pattern of resolution of symptoms when compared with other types of procedures. Second, we only used male Sprague-Dawley rats. Therefore, sex and strain differences in recovery were not tested in our model and affect the generalizability of the findings. Third, we did not evaluate whether the pattern of recovery after second surgical insult would be similar to the initial surgery.^17^ Fourth, it is possible that a reduction in social exploration after surgery reflects an overall reduction in activity and increase in pain-related behaviors, as observed in the locomotor and nociceptive tests, respectively. Fifth, we did not study the effect of a short-term postoperative treatment of aspirin and EPA. Finally, we did not evaluate the effect of docosahexaenoic acid in our model. It is possible to speculate that levels of AT-RvD1 would have been higher if animals would have been treated with docosahexaenoic acid.

In conclusion, we showed that objectively measuring resolution of sickness behavior may be a novel indicator of postoperative recovery. We also demonstrated that aspirin + EPA can improve recovery. Although we observed that aspirin + EPA had not significantly influenced resolvin levels in the intestinal mucosa, we cannot rule out their strong modulation on other organs or cells including monocytes/macrophages because we observed a significant impact on serum cytokine levels.

## Disclosures

The authors have no conflicts of interest to declare.

This work was supported by the University of Texas Rising STARS Award (P.M.G.) and National Institutes of Health grant P30CA016672 (MD Anderson Cancer Center) and from institutional and/or departmental sources (J.P.C.).
